# Optimization of cytarabine (ARA-C) therapy for acute myeloid leukemia

**DOI:** 10.1186/2162-3619-2-20

**Published:** 2013-08-06

**Authors:** Richard L Momparler

**Affiliations:** 1 Département de pharmacologie, Université de Montréal and Service d’hématologie/oncologie, CHU-Saint-Justine, 3175 Cote Sainte-Catherine, Montréal QC H3T 1C5, Canada

**Keywords:** Cytarabine, Cytosine arabinoside, Acute myeloid leukemia, Pharmacology, Drug resistance

## Abstract

Cytarabine (cytosine arabinoside) is one of the most effective drugs for the treatment of acute myeloid leukemia. The standard dose of cytarabine used to treat this leukemia is 100 mg per square meter. In an attempt to improve the effectiveness of cytarabine against acute myeloid leukemia, a high-dose treatment (3,000 mg per square meter) was introduced into therapy. The side effects of high-dose cytarabine was a major concern, especially its neurological toxicity. A review of recent clinical trials indicates that this high-dose cytarabine can be replaced by the intermediate-dose of 1,000 mg per square meter without loss of efficacy and with less toxicity. This is an important step to improve the efficacy of cytarabine for the treatment of acute myeloid leukemia. Despite the improvements in the therapy for this leukemia, the current overall survival rate for adult patients is less than 30%. To optimize the cytarabine therapy, it is important to determine how some leukemic stem cells survive treatment. Preclinical data suggest that survival of the leukemic stem cells could be due to the long 12 hour interval between infusions of cytarabine, which permits some leukemic cells to escape its S phase specific action. Among the other factors that can lead to leukemic cell survival are the high levels in the liver and spleen of cytidine deaminase, the enzyme that inactivates cytarabine and drug resistance due to deficiency in deoxycytidine kinase, the enzyme that activates the prodrug, cytarabine. Several approaches are proposed in this commentary to overcome these impediments with the goal of increasing the effectiveness of cytarabine for the treatment of acute myeloid leukemia.

## Introduction

Cytarabine (cytosine arabinoside, ARA-C) has been used for the treatment of acute myeloid leukemia (AML) for approximately 40 years [1]. One of the first standard dose-schedules (SD) of ARA-C was 100 mg per square meter per day administered as a continuous i.v. infusion for 7 days [1].

The rationale for the use of HD-ARA-C for the treatment of AML was first proposed in 1974 [2]. High-dose (HD) ARA-C was introduced into clinical therapy for AML in 1979 and the early 1980s [3].

Several investigators performed pilot studies in patients with AML using intensive doses of ARA-C and reported several positive results [4-6]. The most comprehensive clinical study was published in 1983 by Herzig and colleagues. They determined that the maximal tolerated dose (MTD) of ARA-C was 3,000 mg per square meter administered as a one-hour infusion every 12 hours for 6 days [6]. The MTD of a drug is not necessarily its most effective therapeutic dose. One of the major concerns of this HD-ARA-C was its toxicity. In addition to myelosuppression, severe neurologic toxicity was also observed in some patients treated with HD-ARA-C [7]. This CNS toxicity was dose-related because it was more severe at the higher ARA-C dose of 4,500 mg per square meter [6]. The pathogenesis of this CNS toxicity remains to be clarified and may be due to the formation of minor metabolites of ARA-C, such as ARA-C-diphosphocholine. Normal cells treated with ARA-C can synthesize significant amounts of ARA-C-diphosphocholine [8], which can interfere with the function of the normal metabolite cytidine diphosphocholine. In support of this hypothesis is the interesting data on the use cytidine diphosphocholine (citicoline) for its “brain-repair” action in the treatment of stroke [9,10].

Due to the concern of the severe side effects of HD-ARA-C observed in some patients with AML, clinical investigators initiated studies using an intermediate dose (ID) of ARA-C in the range of 1,000 to 2,000 mg per square meter [11]. In a recent commentary by Löwenberg entitled: “Sense and nonsense of high-dose cytarabine for acute myeloid leukemia,” he states that the data from several clinical trials indicate that it does not make sense to use HD-ARA-C and that it can be replaced by ID-ARA-C with similar clinical efficacy but less toxicity [12]. Investigations of the metabolism of ARA-C to its active inhibitor, ARA-CTP, by kinases in myeloid leukemic cells from treated patients support this conclusion [13]. This latter study showed that at the 1,000 mg per square meter dose of ARA-C, maximal formation of ARA-CTP is achieved and not increased by the use of the 3,000 mg per square meter dose. During the infusion of HD-ARA-C the steady state plasma level of ARA-C is >100 μM [14]. The enzymes involved in the activation of ARA-C are completely saturated at the lower dose. This is an interesting example of how *in vitro* studies on leukemic cells from patients can provide useful guidelines to optimize the dose of ARA-C for the treatment of AML.

The replacement of HD-ARA-C with ID-ARA-C to improve its therapeutic index is an important advancement for the treatment of AML. The next key question to consider is whether there are other ways to further improve the effectiveness of ARA-C therapy for AML. To make further progress it is important to determine why patients with AML relapse after treatment with ARA-C. In Table 1 is a list of different mechanisms by which leukemic stem cells can possibley “escape” ARA-C treatment and suggestions to overcome these potential impediments to improve the effectiveness of chemotherapy.

### Duration of interval between ARA-C infusions

The long 12-hour interval between the one-hour infusions of HD-ARA-C provides a window for leukemic stem cells to escape its therapeutic action. Because ARA-C has a short plasma half-life [7], 3 hours after the end of the infusion its concentration falls below the therapeutic range. Cell kinetic analysis shows that the S phase of AML cells from some patients can be as short as 8 h [15]. Because ARA-C is an S phase specific agent, some leukemic cells can progress through this phase during the 12-hour interval with minimal or no exposure to cytotoxic concentrations. The original model for intense dose ARA-C for the treatment of AML used a 6-hour interval between drug infusions to reduce the probability of leukemic cells passing through the S phase without exposure to cytotoxic concentrations of this analogue [2]. Laboratory studies using a colony assay show that reduction of the interval between ARA-C exposures to less than 12 hours increases its cytotoxic action against human myeloid leukemic cells [16].

### Drug resistance to ARA-C

Drug resistance to ARA-C can occur by several different mechanisms [17]. ARA-C is a prodrug that is activated by phosphorylation by deoxycytidine (CdR) kinase. Because the human CdR kinase gene is located on chromosome 4, there are two copies of the gene in the cell [18]. Complete drug resistance due to a deficiency in CdR kinase is rare because it requires the gene inactivation of both alleles. It is important to determine if relapse after ARA-C therapy is due to a deficiency in CdR kinase. A simple *in vitro* drug sensitivity test can be performed on blood leukemic blasts. The leukemic cells are incubated with radioactive thymidine and the inhibition of its incorporation into the DNA by ARA-C is determined [19]. Leukemic cells that show some deficiency in CdR kinase show less inhibition of DNA synthesis by ARA-C. We used this *in vitro* test to detect CdR kinase deficiency in leukemic cells from patients treated with the related CdR analogue, 5-aza-2’-deoxycytidine (decitabine) [19,20]. Resistance to ARA-C due to deficiency of CdR kinase in AML cells has also been reported by other investigators [18].

One interesting approach to overcome the problem of ARA-C drug resistance due to a deficiency in CdR kinase is to use 3-deazauridine (3-DU) [21]. 3-DU is a competitive inhibitor of CTP synthetase that reduces the intracellular levels of CTP and dCTP. Wild type leukemic cells can escape the cytotoxic action of 3-DU by using CdR kinase to phosphorylate the CdR present in the extracellular fluid. However, leukemic cells deficient in CdR kinase die due to “dCTP starvation” when treated with 3-DU [21]. The drug-resistant leukemic cells are more sensitive to the chemotherapeutic action of 3-DU than the wild type leukemic cells [20,21]. 3-DU also enhances the cytotoxic action of ARA-C on AML cells by increasing it incorporation into DNA [22].

Another mechanism of drug resistance to ARA-C is due to an increase in the intracellular level of dCTP in leukemic cells [23]. dCTP has a dual action to antagonize the action of ARA-C. First, it acts as a natural competitive inhibitor of the incorporation of ARA-CTP into DNA. Second, dCTP is a feedback inhibitor of CdR kinase that reduces the phosphorylation of ARA-C. As discussed above, 3-DU can reverse both these effects.

The deamination of ARA-C to uracil arabinoside by cytidine (CR) deaminase results in a complete loss of antineoplastic activity. Cells that overexpress CR deaminase show signs of drug resistance to ARA-C [24,25]. We detected an increased expression of CR deaminase in leukemic blasts after treatment with decitabine [19]. Tetrahydrouridine, a potent inhibitor of CR deaminase, can be used to overcome this type of drug resistance [26,27].

### Inactivation of ARA-C by CR deaminase in the liver and spleen

The liver and spleen contain very high levels of CR deaminase [28] that provide a biochemical sanctuary in these organs for leukemic cells due to the deamination of ARA-C to very low concentrations with minimal or no antileukemic activity [27]. This event probably occurs frequently when SD-ARA-C is used because the plasma level of ARA-C is low. Tetrahydrouridine can be used to enhance the antineoplastic action of CdR analogues on tumor cells present in the liver and spleen [27].

### ARA-C can block progression of some leukemic cells from G1 to S phase

The regulation of the progression of cells from G1 to S phase is a complex event that involves a series of activations of different cyclins by phosphorylation [29]. The inhibition of DNA replication by ARA-C in leukemic cells at this G1/S phase check point can lead to a block in the progression of some leukemic cells into S phase. Evidence for this rare event comes from studies that compare the antineoplastic activity of ARA-C and decitabine in leukemic cells [30]. Both these agents are deoxycytidine analogues and S phase specific agents because their antileukemic action is due to their incorporation into DNA. However, their mechanisms of action are different; ARA-C inhibits DNA synthesis whereas decitabine inhibits DNA methylation. Because both of these agents target the same cohort of leukemic cells in the S phase, they should produce an identical loss of survival at equal exposure times and therapeutic concentrations. However, colony assays show that more myeloid leukemic cells survive after ARA-C treatment than after decitabine [30]. Additionally, in a mouse model of L1210 leukemia using the same duration of infusion, decitabine cured the mice, but ARA-C at its MTD did not [31]. The partial G1/S phase block by ARA-C can permit a small fraction of the leukemic cells to escape its chemotherapeutic action. There are two possible approaches than could be used to overcome this problem. First, it is possible that some leukemic cells may recover from this block when an intermittent ARA-C treatment is used rather than a continuous exposure. Second, it is possible to replace ARA-C with decitabine, whose epigenetic action does not block progression of the G1 cell into S phase [32]. It would be interesting to use a colony assay to compare the antineoplastic activity of ARA-C and decitabine on leukemic cells to identify the patients that would benefit from decitabine therapy.

### High frequency of leukemic stem cells

Research shows that upon analysis of leukemic cells from patients with AML, only a small fraction of the cells function as leukemic stem cells [33]. Long-term survival of patients with leukemia is dependent on the complete eradication of the leukemic stem cells by chemotherapy. It has been reported that a high stem cell frequency in AML and acute lymphoblastic leukemia predicts a poor survival rate [34,35]. We do not fully understand why AML patients with adverse cytogenetics are less responsive to ARA-C therapy than AML patients with a normal karyotype [1]. One possible explanation is that these high-risk AML patients have a higher frequency of leukemic stem. To completely eradicate the leukemic stem cells with ARA-C in the high-risk AML patients, one has to increase the clinical efficacy of the chemotherapy. One approach to accomplish this objective is to use ARA-C in combination with 3-DU, which enhances the cytotoxic action of ARA-C on AML cells [22]. Another interesting approach is to use epigenetic priming with decitabine prior to SD-ARA-C treatment of patients with AML [36]. This latter approach was used in a phase I study and produced 83% CR in patients with less-than-favorable risk of AML.

### Resistance to apoptosis

The suppression of apoptosis in malignant cells can occur by epigenetic silencing. Aberrant DNA methylation was reported to silence the pro-apoptotic gene, FOXO3 [37] and to down-regulate the TRAIL pathway [38]. Treatment with decitabine can lead to the reactivation of these pro-apoptotic pathways [37,38]. An interesting approach to overcome the problem of ARA-C resistance to apoptosis is to use epigenetic priming with decitabine to reactivate the apoptotic pathway prior to the ARA-C therapy [36]. Mutations in p53 are associated with resistance to ARA-C therapy in patients with AML [39]. This drug resistance is due, in part, to interference with the normal apoptotic pathway in AML cells. Decitabine was shown to be effective against p53-null AML cells and more active than ARA-C [40]. This epigenetic agent can be used to treat AML patients that show resistance to the induction of apoptosis by ARA-C.

## Conclusion

To improve the effectiveness of chemotherapy for the treatment of AML, we have to learn why ARA-C does not completely eradicate all the leukemic stem cells. *In vitro* tests on leukemic cells from AML patients before and after relapse can give insight on how some leukemic cells survive ARA-C therapy. Several approaches can be investigated to improve the efficacy of ARA-C (Table 1). In addition to these approaches, the long interval (4 weeks) between cycles of ARA-C therapy can permit surviving leukemic cells to expand unimpeded, which can also be a source for treatment failure. Recent results indicate that it is possible to use very low (metronomic) doses (1-3X/week) of decitabine with good responses in patients with myelodysplastic syndrome [41]. This low dose decitabine can maintain or increase self-renewal of normal hematopoietic stem cells [42]. An interesting alternative is to use non-toxic genistein to slow the progression of leukemia between cycles of ARA-C [43]. It would be of interest to perform pilot studies using novel ARA-C regimens on cohorts of high-risk AML patients that have a limited life expectancy. These pilot studies to optimize ARA-C therapy can possibly lead to a better understanding of the pharmacology of this analogue and leukemogenesis and lead to an improved survival in patients with AML.

**Table 1:** Possible mechanisms by which leukemic stem cells survive after ARA-C treatment and approaches to optimize therapy

	**Treatment to reduce**	
**Survival mechanisms**	**leukemic cell survival**	**References**
12-h interval between ARA-C infusions too long	reduce interval	[16]
deficiency in CdR kinase	3-DU + ARA-C	[20-22]
increase in dCTP	3-DU + ARA-C	[20-22]
increase CR deaminase	THU + ARA-C	[24,25]
CR deaminase inactivation of ARA-C in liver	THU + ARA-C	[24,25]
block in cell cycle progression G1 to S phase	intermittent ARA-C, DAC	[30]
high frequency of leukemic stem cells	3-DU + ARA-C, DAC prime ARA-C	[22,36]
resistance to apoptosis	DAC prime ARA-C, DAC	[36,40]

*Abbreviations: h* hour, *3-DU* 3-deazauridine, *THU* tetrahydrouridine, *DAC* decitabine.

## Competing interests

The author declares that he has no competing interests.

## Author’s contribution

RLM wrote and approved the final manuscript.

## ADDENDUM

An excellent example of an *in vitro* colony assay on leukemic cells from patients to evaluate the efficacy of chemotherapy is shown in the publication by: Craddock C, Quek L, Goardon N, et al: **Azacitidine fails to eradicate leukemic stem/progenitor cell populations in patients with acute myeloid leukemia and myelodysplasia.***Leukemia.* 2013, **27:**1028-1036.
